# Micronutrient status and growth outcomes in children with cow’s milk allergy: beyond elimination diets

**DOI:** 10.3389/fnut.2026.1886883

**Published:** 2026-06-24

**Authors:** Mariano Di Rosa, Simone Foti Randazzese, Mara De Amici, Daniele Giovanni Ghiglioni, Alessia Marseglia, Gian Luigi Marseglia, Elvira Verduci, Amelia Licari

**Affiliations:** 1 Pediatric Unit, Department of Clinical, Surgical, Diagnostic, and Pediatric Sciences, University of Pavia, Pavia, Italy; 2 Pediatric Clinic, Fondazione IRCCS Policlinico San Matteo, Pavia, Italy; 3 Immuno-Allergology Laboratory of Clinical Chemistry and Pediatric Clinic, Fondazione IRCCS Policlinico San Matteo, Pavia, Italy; 4 SC Pediatria Pneumoinfettivologia, Fondazione IRCCS Ca’ Granda Ospedale Maggiore Policlinico, Milan, Italy; 5 Department of Health Sciences, University of Milan, Milan, Italy; 6 Pediatric Unit, Fondazione IRCCS Ca’ Granda Ospedale Maggiore Policlinico, Milan, Italy

**Keywords:** cow’s milk allergy, elimination diet, growth, micronutrient deficiency, pediatric nutrition

## Abstract

Cow’s milk allergy (CMA) is among the most common food allergies in early childhood, affecting approximately 2–3% of children under 3 years of age. Management requires strict elimination of cow’s milk and dairy products, typically replaced with hypoallergenic formulas. Although essential, this dietary restriction may predispose infants—particularly during the first 2 years of life—to nutritional imbalances. This mini review synthesizes current evidence on micronutrient status and growth outcomes in children with CMA, with emphasis on the impact of dietary management. Across observational studies, vitamin D inadequacy is the most consistently reported abnormality, with insufficiency affecting approximately 30–55% of children and deficiency around 20–25%. Iron deficiency is also frequent, including both overt anemia and subclinical depletion. Lower levels of calcium, vitamin B12, and iodine have been described, generally within reference ranges but suggestive of suboptimal intake. Growth impairment is commonly observed at diagnosis, particularly affecting weight more than linear growth. Appropriate nutritional management, including the use of hypoallergenic formulas (HF) and dietary counselling, is associated with significant catch-up growth. However, an increased risk of being overweight has been reported during follow-up, highlighting the need for balanced nutritional strategies. Key risk factors for nutritional compromise include multiple food allergies, prolonged dietary restriction without adequate substitution, poor adherence to supplementation, and restrictive maternal diets during breastfeeding. Overall, CMA management should extend beyond allergen avoidance to include proactive nutritional surveillance, targeted supplementation, and individualized dietary planning to support optimal growth and long-term health.

## 1 Introduction

Cow’s milk allergy (CMA) is the most common food allergy (FA) in early childhood, affecting approximately 2–3% of children under 3 years of age in high-income countries, with prevalence estimates reaching up to 7.5% depending on diagnostic criteria and study design (1–3). CMA is an immune-mediated adverse reaction to one or more cow’s milk proteins—primarily *α*-lactalbumin, *β*-lactoglobulin, and caseins—resulting in reproducible symptoms after ingestion (4). Three major immunopathological phenotypes are recognized: immunoglobulin E (IgE)-mediated, non-IgE-mediated, and mixed forms (5). IgE-mediated CMA typically presents with rapid-onset manifestations, including urticaria, angioedema, vomiting, respiratory compromise, and anaphylaxis (5, 6), whereas non-IgE-mediated forms involve delayed, predominantly gastrointestinal manifestations such as food protein-induced allergic proctocolitis, food protein-induced enterocolitis syndrome, and food protein-induced enteropathy (7, 8). Mixed forms, including eosinophilic esophagitis, involve both IgE- and cell-mediated immune mechanisms (1). The natural history of CMA is generally favorable, with tolerance developing in 50–80% of children by 3–5 years of age, although persistence is more common in IgE-mediated disease and severe phenotypes (9, 10).

Management relies on strict elimination of cow’s milk proteins, including maternal exclusion during breastfeeding when clinically indicated (10). Extensively hydrolyzed formulas are recommended as first-line treatment, whereas amino acid-based formulas are reserved for severe or refractory cases (3, 11). Although essential for symptom control, elimination diets may increase the risk of nutritional imbalance during infancy, a period characterized by rapid growth, high metabolic demands, and increased vulnerability to nutritional deficiencies (12–16).

Cow’s milk and dairy products are important dietary sources of several nutrients relevant to growth and development, including calcium, vitamin B12, iodine, and high-quality protein (17). Consequently, children with CMA following prolonged elimination diets may be at risk of inadequate intake and/or biochemical deficiencies of several nutrients (18–20).

Calcium and vitamin D are essential for bone mineralization and skeletal homeostasis. Calcium requirements increase from approximately 200–260 mg/day during the first year of life to nearly 700 mg/day during the second year, while vitamin D supplementation of 400 IU/day is recommended throughout infancy regardless of feeding modality (20, 21). Iron is essential for hemoglobin synthesis, cellular metabolism, and neurodevelopment, with requirements increasing substantially during late infancy as endogenous iron stores decline, reaching approximately 11 mg/day between 6 and 12 months of age (20, 22). Vitamin B12 and iodine are likewise critical during infancy, supporting DNA synthesis, erythropoiesis, neurodevelopment, and thyroid hormone production. Recommended vitamin B12 intake is approximately 0.4–0.5 μg/day, whereas iodine requirements range from about 90 μg/day during the first year of life to 90–100 μg/day during the second year; notably, dairy products constitute a major dietary source of both nutrients in many populations (20, 23, 24). Zinc and selenium further contribute to immune regulation, epithelial integrity, and metabolic homeostasis (20, 25, 26).

Breastfeeding remains the preferred feeding strategy because of its nutritional and immunological benefits (27–29). However, maternal elimination diets require appropriate nutritional counselling and, when necessary, supplementation to ensure adequate maternal and infant nutrient status (19, 30). When breastfeeding is not feasible, extensively hydrolyzed or amino acid-based formulas represent the standard nutritional alternative (31, 32). Careful planning of complementary feeding is also required to maintain nutritional adequacy while ensuring safe allergen avoidance (33–35).

Despite increasing recognition of these nutritional challenges, evidence regarding micronutrient status and growth outcomes in children with CMA remains heterogeneous. This review synthesizes current evidence on micronutrient status and growth outcomes in children with CMA, with particular focus on dietary management, nutritional monitoring, and clinical implications.

## 2 Literature search strategy

A structured literature search was performed in PubMed, Scopus, and MEDLINE to identify relevant studies published between January 2016 and January 2026. Search terms included combinations of “cow’s milk allergy,” “cow’s milk protein allergy,” “elimination diet,” “nutritional status,” “micronutrient deficiency,” “vitamin D,” “calcium,” “iron,” “vitamin B12,” “iodine,” “growth,” and “children.” Observational studies, prospective and retrospective cohorts, case–control studies, clinical guidelines, position papers, and relevant reviews addressing nutritional status, micronutrient intake or biomarkers, growth outcomes, and dietary management in infants and children with CMA were considered. Studies focusing exclusively on adult populations, non-CMA food allergies without separate CMA data, or non-nutritional outcomes were excluded. As this was a narrative mini review rather than a systematic review, protocol registration, PRISMA reporting, formal risk-of-bias assessment, and standardized data extraction procedures were not performed. Given the heterogeneity of study designs, populations, diagnostic criteria, and reported outcomes, a narrative synthesis was considered more appropriate than a formal systematic review or meta-analysis.

## 3 Micronutrient status in children with CMA

### 3.1 Vitamin D

Vitamin D is the most consistently reported micronutrient inadequacy in children with CMA. Across available studies, vitamin D insufficiency has been reported in approximately 30–55% of children, whereas overt deficiency affects nearly 20–25% (36, 37). However, these findings should be interpreted in the context of the broader epidemiology of pediatric hypovitaminosis D. Several studies have reported high rates of vitamin D deficiency and insufficiency in the general pediatric population, ranging on average between 40 and 75%, even in developed countries (38). Therefore, vitamin D inadequacy in CMA likely reflects the combined contribution of disease-specific factors and the high baseline prevalence of pediatric vitamin D insufficiency.

Silva et al. (36) reported lower mean vitamin D levels in CMA infants compared with controls (30.93 ± 12.33 vs. 35.29 ± 10.74 ng/mL; *p* = 0.041) and higher deficiency prevalence (20.3% vs. 8.2%; *p* = 0.049). Gamal et al. (39) observed markedly lower median levels (20.0 vs. 40.0 ng/mL; *p* < 0.001). Similarly, Pandiaraja et al. (40) reported lower median levels in CMA compared with controls (47.5 vs. 62.5 nmol/L; *p* = 0.030), and higher insufficiency rates (55.6% vs. 30.8%; *p* = 0.091).

Overall, vitamin D inadequacy is likely multifactorial, reflecting dietary restriction, inadequate supplementation, breastfeeding without supplementation, and reduced intake of fortified products. However, interpretation of the available evidence is complicated by potential confounding factors, including age, seasonality, sunlight exposure, dietary habits, and adherence to vitamin D supplementation. Consequently, the extent to which CMA independently contributes to vitamin D deficiency remains uncertain and warrants further investigation in prospective studies specifically designed to control for these variables.

### 3.2 Calcium

Calcium levels are often lower in CMA but typically remain within reference ranges. Gamal et al. (39) reported lower median serum calcium in CMA children compared with controls (8.5 vs. 9.9 mg/dL; *p* ≤ 0.001). Similarly, Maleknejad et al. (19) observed slightly higher calcium concentrations in breastfed compared with formula-fed infants (10.17 ± 0.47 vs. 9.94 ± 0.37 mg/dL; *p* = 0.036).

Serum calcium is tightly regulated and does not reflect intake or bone mineralization. The main concern is reduced bone mineral accrual. Long-term dairy exclusion has been associated with reduced bone mineral density and delayed skeletal maturation (41).

Accordingly, clinical management should prioritize regular assessment of dietary calcium intake rather than reliance on serum levels alone. Given the variable bioavailability of non-dairy sources, the use of fortified foods or nutritionally complete formulas is often required, especially in infants and young children.

### 3.3 Iron

Iron deficiency is common and often associated with anemia. Gamal et al. (39) reported significantly lower median iron levels (22 vs. 70 μg/L; *p* ≤ 0.001), hemoglobin (8.5 vs. 12.35 g/dL; *p* ≤ 0.001), and mean corpuscular volume (61 vs. 81.4 fL; *p* ≤ 0.001) compared with controls. Prospective data show persistent abnormalities, with lower ferritin (*p* = 0.009) and higher prevalence of functional iron deficiency (*p* = 0.027) (42).

The pathophysiology is multifactorial, including reduced dietary iron intake, occult gastrointestinal blood loss (particularly in non-IgE-mediated gastrointestinal forms), and inflammation-mediated impairment of iron absorption and mobilization (37, 39, 42).

Systematic biochemical monitoring and early correction of iron deficiency should be integral components of CMA management, particularly in infants with gastrointestinal involvement or suboptimal growth.

### 3.4 Vitamin B12

Vitamin B12 deficiency has been less extensively investigated but remains clinically relevant. Jardim-Botelho et al. (42) reported deficiency in 12.5% of CMA infants vs. 0% of controls. Longitudinally, CMA infants exhibited a significantly smaller increase in serum vitamin B12 concentrations over 18 months compared with controls (*p* = 0.001), indicating persistent vulnerability despite dietary management.

Risk is highest in breastfed infants with maternal dietary restriction. This has important clinical implications, as vitamin B12 deficiency in infancy can lead to hematologic abnormalities and neurological complications, including hypotonia, developmental delay, seizures, and potentially irreversible neurodevelopmental impairment. Consequently, maternal dietary assessment and targeted supplementation should be considered in cases of prolonged or highly restrictive elimination diets (43).

### 3.5 Iodine

Jardim-Botelho et al. (42) reported lower urinary iodine concentrations in CMA infants compared with controls (198 vs. 296 μg/L; *p* = 0.034) and smaller increase over time (*p* < 0.001). Although values remained within reference ranges, findings indicate a relative deficit. Given that iodine assessment is not standard in clinical practice, deficiency may remain underdiagnosed in this population.

### 3.6 Other micronutrients

Evidence for other micronutrients remains limited and heterogeneous. Gamal et al. (39) reported no significant difference in zinc levels between CMA children and controls (66 vs. 71 μg/dL; *p* = 0.228). In the same study, magnesium levels were slightly lower in CMA children (2.0 vs. 2.1 mg/dL; *p* < 0.001), although the absolute difference was minimal and of uncertain clinical significance. Similarly, Maleknejad et al. (19) observed no differences in zinc, phosphorus, or magnesium concentrations between feeding groups, while reporting higher selenium levels in formula-fed compared with breastfed infants with CMA (87.42 ± 25.63 vs. 64.65 ± 19.08 μg/dL; *p* = 0.0001). Importantly, all reported values remained within age-appropriate reference ranges.

These findings suggest that statistically significant differences in selected micronutrients may reflect variations in dietary intake, feeding modality, or nutritional exposure rather than clinically relevant deficiency states. At present, there is insufficient evidence to support routine biochemical screening or empirical supplementation of zinc, magnesium, or selenium in otherwise well-managed children with CMA. Nevertheless, targeted evaluation may be warranted in selected high-risk patients, including those with multiple FA, chronic gastrointestinal symptoms, malabsorption, poor growth, or prolonged restrictive diets. Further studies are needed to determine whether the observed biochemical differences have meaningful implications for long-term clinical outcomes.

The main micronutrient concerns reported in children with CMA, together with relevant risk factors and practical monitoring considerations, are summarized in Table 1.

**Table 1:** Main micronutrient concerns in children with cow’s milk allergy: evidence, risk factors, and clinical considerations.

Nutrient	Evidence in CMA	Children at higher risk	Clinical considerations
Vitamin D	Most consistently reported micronutrient inadequacy; insufficiency reported in ~30–55% of children and deficiency in ~20–25%	Breastfed infants without supplementation; prolonged dairy exclusion; poor adherence to supplementation	Ensure routine vitamin D supplementation during infancy; consider assessment of serum 25(OH)D in high-risk children or when adherence/intake is uncertain; treat deficiency according to local recommendations
Calcium	Reduced dietary intake may occur despite normal serum calcium concentrations	Inadequate consumption of fortified substitutes; prolonged dairy exclusion; multiple FA	Prioritize assessment of dietary calcium intake rather than serum calcium alone; promote use of fortified foods or nutritionally complete HF; consider supplementation when dietary intake is inadequate
Iron	Iron deficiency, functional iron deficiency, and iron-deficiency anemia are frequently reported	Non-IgE-mediated gastrointestinal CMA; occult gastrointestinal blood loss; restrictive complementary feeding practices	Periodically assess hemoglobin, ferritin, and transferrin saturation in at-risk children; encourage iron-rich complementary feeding and treat confirmed deficiency promptly
Vitamin B12	Deficiency has been reported; persistent vulnerability may occur despite dietary management	Breastfed infants of mothers following restrictive elimination diets	Assess maternal dietary intake and vitamin B12 status when clinically indicated; consider maternal and/or infant supplementation in cases of inadequate intake or confirmed deficiency
Iodine	Lower urinary iodine concentrations reported, generally within reference ranges but suggestive of suboptimal iodine status	Maternal dairy exclusion; breastfeeding during maternal elimination diets	Ensure adequate maternal iodine intake; consider iodine assessment and supplementation in selected high-risk children and breastfeeding mothers
Zinc	Limited and heterogeneous evidence; concentrations are generally within reference ranges	Multiple FA; poor dietary diversity; malabsorption	Routine screening is not supported by current evidence; consider targeted assessment in children with poor growth, chronic gastrointestinal symptoms, or highly restrictive diets
Selenium	Differences according to feeding modality have been reported, although concentrations generally remain within reference ranges	Maternal dietary restriction; multiple food exclusions	Promote adequate dietary intake; reserve biochemical assessment and supplementation for selected high-risk children

25(OH)D, 25-hydroxyvitamin D; CMA, cow’s milk allergy; FA, food allergy; HF, hypoallergenic formula; IgE, immunoglobulin E.

## 4 Growth outcomes and feeding modalities

Growth impairment is commonly reported at diagnosis in children with CMA. Gamal et al. (39) reported weight <3rd percentile in 61.8%, BMI < 3rd percentile in 49%, and height <3rd percentile in 24.5% (all *p* < 0.001).

Prospective studies show improvement with nutritional management. In the cohort by Jardim-Botelho et al. (42), children with CMA showed a significantly greater increase in weight-for-age and length-for-age z-scores over time compared with controls (*p* = 0.008 and *p* = 0.001, respectively). Importantly, baseline anthropometric differences between groups were not statistically significant.

Similarly, Taveira et al. (44) reported significant improvements in all anthropometric indices after HF treatment (*p* < 0.001). However, this improvement was accompanied by a significant increase in the prevalence of overweight/obesity risk (*p* < 0.001), with the proportion rising from 11.38 to 23.35%.

Furthermore, comparative data indicate distinct micronutrient profiles between breastfed and formula-fed infants with CMA. In the study by Maleknejad et al. (19), breastfed infants had higher mean serum calcium levels than formula-fed infants (10.17 ± 0.47 vs. 9.94 ± 0.37 mg/dL; *p* = 0.036), whereas formula-fed infants showed significantly higher iron (70.70 ± 26.02 vs. 43.27 ± 20.69 μg/dL; *p* = 0.001) and selenium concentrations (87.42 ± 25.63 vs. 64.65 ± 19.08 μg/dL; *p* = 0.000). No significant differences were observed for vitamin D, zinc, phosphorus, or magnesium, indicating that most micronutrient levels remained comparable between groups. Importantly, all measured values were within normal reference ranges, suggesting that these differences are primarily biochemical rather than indicative of overt deficiency.

Beyond nutrient composition, breastfeeding provides well-established benefits that extend beyond micronutrient status. Human milk contains a complex array of bioactive compounds, including secretory IgA, lactoferrin, lysozyme, cytokines, growth factors, and human milk oligosaccharides, which contribute to immune maturation, gut barrier integrity, and the development of a healthy intestinal microbiome. These properties are important for overall infant health and may be particularly relevant during early life, when immune and gastrointestinal systems are rapidly developing (27–29).

However, complementary evidence shows that breastfed infants with CMA are at higher risk of vitamin D inadequacy compared with formula-fed peers (*p* = 0.002), likely reflecting the low intrinsic vitamin D content of human milk (36).

Taken together, these findings suggest that feeding modality influences selected nutritional biomarkers but does not inherently determine nutritional adequacy when appropriately managed. Breastfeeding remains the preferred feeding strategy for infants with CMA because of its established nutritional, immunological, and developmental benefits. Nevertheless, successful breastfeeding in this setting requires appropriate nutritional counselling, maternal dietary supervision when elimination diets are implemented, and adherence to recommended infant supplementation practices. The choice between breastfeeding and hypoallergenic formula should therefore be individualized according to clinical severity, maternal nutritional status, family preferences, and the feasibility of ongoing nutritional monitoring.

## 5 Discussion

The available evidence indicates that children with CMA may be at increased risk of micronutrient inadequacy and growth alterations, although the magnitude and clinical relevance of these abnormalities vary considerably across studies. Importantly, nutritional outcomes appear to depend not only on allergen avoidance itself, but also on the adequacy of dietary substitution, supplementation practices, feeding modality, caregiver adherence, and access to specialized nutritional follow-up.

Several clinical and environmental factors have been associated with a higher risk of nutritional compromise. Older children who continue prolonged cow’s milk avoidance without adequate intake of fortified alternatives appear particularly vulnerable, with vitamin D insufficiency reported in up to 83% of cases (37). Disease phenotypes may also influence nutritional status. Pandiaraja et al. (40) observed significantly lower serum vitamin D concentrations in children with IgE-mediated CMA compared with non-IgE-mediated forms (39.0 vs. 53.0 nmol/L; *p* = 0.041), suggesting that more restrictive dietary behaviors, greater disease persistence, or differences in inflammatory burden may contribute to impaired micronutrient status in selected phenotypes.

At the same time, several potentially modifiable factors deserve consideration. Poor adherence to supplementation is frequently observed in clinical practice, with objective compliance often substantially lower than caregiver-reported adherence. Maternal elimination diets during breastfeeding may further increase the risk of vitamin B12, iodine, and vitamin D inadequacy, particularly when nutritional counseling is limited or supplementation is inadequate. Socioeconomic determinants, including access to specialized formulas, dietary counseling, and multidisciplinary care, may additionally influence nutritional outcomes and contribute to disparities in long-term management (37, 40, 43).

Current evidence also highlights important methodological limitations. Most available studies are observational, involve relatively small sample sizes, and differ substantially regarding diagnostic criteria for CMA, age distribution, feeding practices, supplementation protocols, and biochemical cut-offs for micronutrient deficiency. Moreover, some abnormalities reported in children with CMA—particularly vitamin D insufficiency—may partly reflect the high baseline prevalence of these deficiencies in the general pediatric population rather than CMA-specific mechanisms alone. Consequently, distinguishing disease-related nutritional risk from broader pediatric nutritional epidemiology remains challenging.

These considerations reinforce the importance of proactive and individualized nutritional monitoring. Vitamin D supplementation at 400 IU/day should be routinely recommended during infancy, while biochemical assessment may be particularly relevant in high-risk children, including those with prolonged elimination diets, poor adherence, multiple food allergies, or impaired growth. Iron status should be periodically evaluated using hemoglobin, ferritin, and transferrin saturation, especially in children with gastrointestinal involvement or restrictive complementary feeding patterns. Calcium adequacy should primarily be assessed through dietary evaluation, as serum calcium concentrations are tightly regulated and may not accurately reflect body stores or bone mineralization (20, 21, 45). In selected severe cases, assessment of bone health may also be considered.

Maternal nutritional status represents another critical but often underrecognized aspect of CMA management. In breastfeeding mothers following prolonged elimination diets, assessment and supplementation of vitamin B12 and iodine should be considered, with infant evaluation when clinically indicated (46). These findings further support the need for multidisciplinary management integrating pediatricians, allergists, and dietitians to optimize nutritional adequacy while maintaining effective allergen avoidance.

An additional key aspect is the periodic reassessment of tolerance acquisition. Prolonged unnecessary dietary restriction may itself contribute to nutritional compromise and negatively affect quality of life. The milk ladder approach, based on gradual reintroduction of cow’s milk proteins from extensively heated to less processed forms, is increasingly used in clinical practice (47). Approximately 70–80% of children with IgE-mediated CMA tolerate baked milk products, and early introduction of baked forms may promote tolerance acquisition in selected patients (48). Nevertheless, timing and modality of reintroduction should be individualized according to disease phenotype and performed under specialist supervision in IgE-mediated CMA because of the risk of systemic reactions (49). Beyond immunological benefits, successful dietary reintroduction may improve intake of calcium, vitamin B12, iodine, and high-quality protein, with potentially favorable implications for bone and metabolic health.

**Figure 1:**
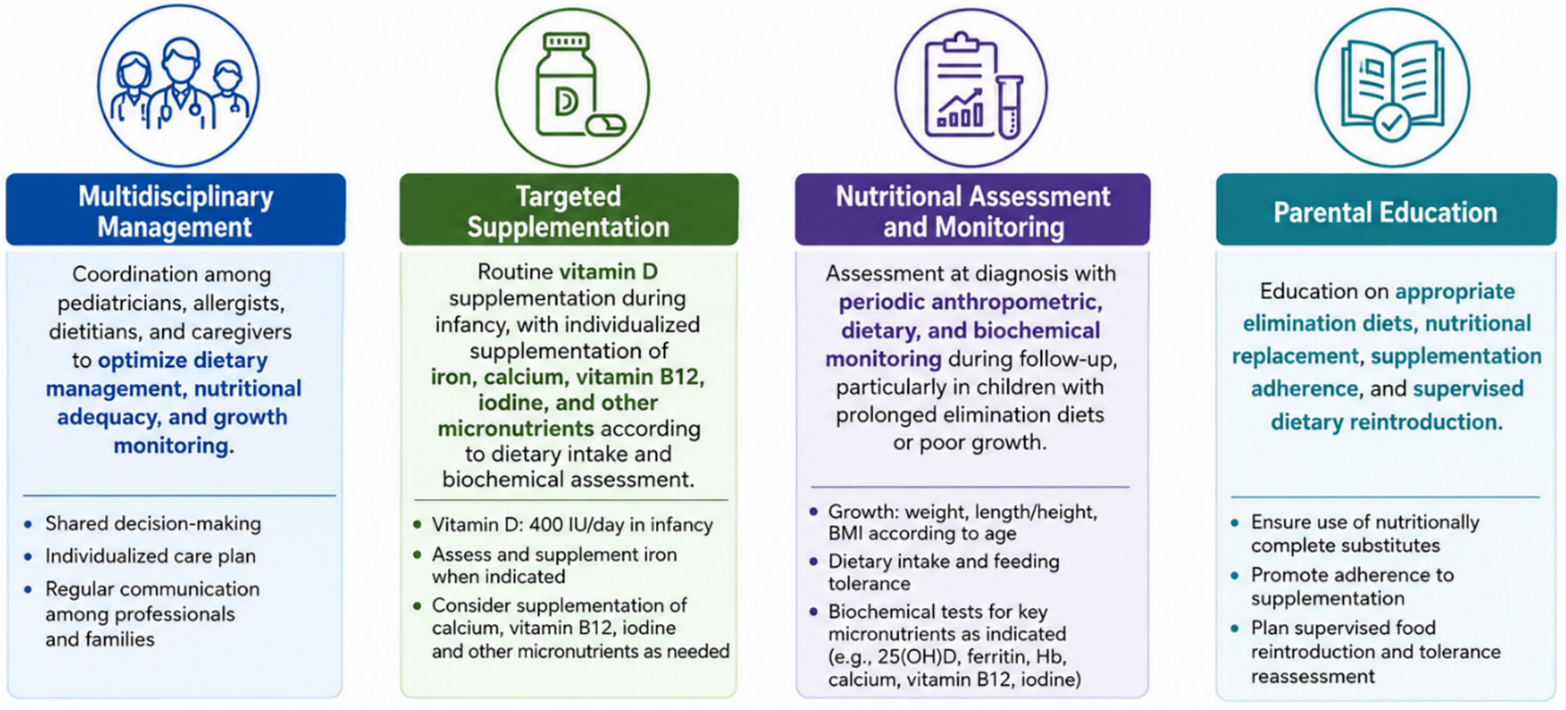
Multidisciplinary nutritional management of children with cow’s milk allergy. Structured management of children with cow’s milk allergy should extend beyond allergen avoidance to include nutritional assessment, targeted micronutrient supplementation, growth monitoring, caregiver education, and supervised dietary reintroduction. A coordinated multidisciplinary approach may reduce the risk of micronutrient deficiencies and growth impairment associated with prolonged or inadequately substituted elimination diets. 25(OH)D, 25-hydroxyvitamin D; BMI, body mass index; CMA, cow’s milk allergy; Hb, hemoglobin.

Despite increasing recognition of nutritional risks associated with CMA, several important gaps remain. Standardized protocols for micronutrient screening and follow-up are lacking, and long-term outcomes related to growth, neurodevelopment, body composition, and metabolic health remain insufficiently characterized. Future studies should prioritize longitudinal designs evaluating the impact of different dietary strategies, formula compositions, supplementation protocols, and tolerance acquisition pathways on long-term nutritional outcomes. Greater emphasis should also be placed on personalized nutritional management and multidisciplinary care models aimed at balancing effective allergen avoidance with optimal nutritional support and parental education (Figure 1).

Overall, current evidence suggests that children with CMA may be particularly vulnerable to micronutrient inadequacy and early growth alterations, especially in the context of prolonged or inadequately substituted elimination diets. However, available evidence indicates that many nutritional complications may be preventable through early diagnosis, appropriate dietary replacement, targeted supplementation, and structured nutritional surveillance. Management of CMA should therefore extend beyond allergen avoidance alone to include regular anthropometric and biochemical assessment, individualized dietary planning, caregiver education, and periodic reassessment of tolerance acquisition, while also monitoring for excessive weight gain during nutritional recovery. Nevertheless, the predominantly observational nature of the available studies and the heterogeneity of reported outcomes highlight the need for further prospective research to better define long-term nutritional risks and optimal monitoring strategies.
